# Ectopic expression of a novel CD22 splice-variant regulates survival and proliferation in malignant T cells from cutaneous T cell lymphoma (CTCL) patients

**DOI:** 10.18632/oncotarget.3720

**Published:** 2015-03-30

**Authors:** Ieva Bagdonaite, Hans H. Wandall, Ivan V. Litvinov, Claudia Nastasi, Jürgen C. Becker, Sally Dabelsteen, Carsten Geisler, Charlotte M. Bonefeld, Qian Zhang, Mariusz A. Wasik, Youwen Zhou, Denis Sasseville, Niels Ødum, Anders Woetmann

**Affiliations:** ^1^ Department of International Health, Immunology and Microbiology, University of Copenhagen, Copenhagen, Denmark; ^2^ Copenhagen Center for Glycomics, Department of Cellular and Molecular Medicine, University of Copenhagen, Copenhagen, Denmark; ^3^ Division of Dermatology, McGill University Health Centre, Montreal, Quebec, Canada; ^4^ General Dermatology, Medical University of Graz, Austria; ^5^ Department of Oral Medicine and Pathology, School of Dentistry, University of Copenhagen, Copenhagen, Denmark; ^6^ Department of Pathology and Laboratory Medicine, University of Pennsylvania, Philadelphia, USA; ^7^ Department of Dermatology and Skin Science, University of British Columbia, Vancouver, BC, Canada

**Keywords:** CTCL, CD22

## Abstract

CD22 is a member of the Sialic acid-binding Ig-like lectin (Siglec) family of lectins described to be exclusively present in B lymphocytes and B cell-derived neoplasms. Here, we describe a novel splice form of CD22 (designated CD22ΔN), which lacks the N-terminal domain as demonstrated by exon-specific RT-PCR and differential recognition by anti-CD22 antibodies. Importantly, CD22ΔN mRNA is expressed in skin lesions from 39 out of 60 patients with cutaneous T cell lymphoma (CTCL), whereas few patients (6 out of 60) expresses full-length, wild type CD22 (CD22wt). In addition, IHC staining of tumor biopsies confirmed the expression of CD22 in CD4^+^ T cells. Moreover, four out of four malignant T cell lines express CD22: Two cell lines express CD22ΔN (MyLa2059 and PB2B) and two express CD22wt (MAC-1 and MAC-2A). siRNA-mediated silencing of CD22 impairs proliferation and survival of malignant T cells, demonstrating a functional role for both CD22ΔN and CD22wt in these cells.

In conclusion, we provide the first evidence for an ectopic expression of CD22 and a novel splice variant regulating malignant proliferation and survival in CTCL. Analysis of expression and function of CD22 in cutaneous lymphomas may form the basis for development of novel targeted therapies for our patients.

## INTRODUCTION

Mycosis fungoides (MF) is the most common form of Cutaneous T Cell Lymphoma (CTCL), representing more than 50% of total cases. CTCL is a non-Hodgkin's lymphoma involving mature skin-resident T lymphocytes [1, 2]. Prognosis is favorable for early-stage patients, with life span approaching that of healthy age-matched controls. However, it is less favorable for patients with more advanced disease stages. Failure of the immune system leading to sepsis or opportunistic infections is the usual cause of disease-related death [2]. Various skin-directed therapies are available for MF patients, and generally patients with patch and plaque stages respond well to topical treatment and other skin-directed modalities. In contrast, treatment of advanced disease is difficult as most patients become resistant to therapy [1]. Thus, it is important to understand the biology of MF and find new targets to enhance therapeutic efficacy.

Malignant transformation may lead to developmental aberrations resulting in mixed phenotype characterized by expression of cell surface markers indicative of several lymphoid lineages [3]. Our previous studies of novel markers for cutaneous T-cell lymphomas revealed ectopic expression of B-lymphoid tyrosine kinase (BLK) both *in vitro* in CTCL cell lines as well as MF lesional skin [4]; this observation was recently confirmed in independent studies [5, 6]. Importantly, BLK in CTCL is functional, activated and involved in the spontaneous proliferation of malignant T cells [4]. This notion was unexpected as BLK is normally expressed exclusively in B cells and thymocytes [7]. This discovery prompted us to screen for additional proteins physiologically restricted to the B-cell linage in MF.

CD22 is a member of the Siglec (sialic acid-binding Ig-like lectin) family of lectins and the immunoglobulin superfamily [8]. CD22 expression has been exclusively described in B cells [9] until recently when ectopic expression of CD22 was demonstrated in lung cancer cells [10]. During B cell development CD22 is present in pro-B and pre-B cells, but at these stages the expression is restricted to the cytoplasm. In mature B cells CD22 is expressed on the surface, however, eventually such expression is lost when B cells differentiate into plasma cells [11]. In lymphoid tissues CD22 is expressed in follicular mantle and marginal zone B cells, but only weakly in germinal center B cells [12]. CD22 functions as a negative co-receptor in B cell signaling and prevents B cells from overstimulation upon activation [13]. Furthermore, CD22 ligand binding is implicated in the survival of both normal and malignant B cells [14]. There are 2 splice variants of CD22; CD22α (130 kDa) and CD22β (140 kDa) with 5 and 7 extracellular immunoglobulin (Ig) domains, respectively. The N-terminal domain of CD22 is a V-set Ig domain, while the remaining extracellular domains are C2-set Ig domains. CD22α lacks domains 3 and 4 [12, 15, 16]. The two distal extracellular domains are responsible for ligand binding [14] with high specificity to α2,6-sialylated ligands on N-linked glycans [17]. CD22 predominantly exists as a monomer of CD22β [12], but it can also be found as a heterodimer together with CD22α [18].

Here we report that CD22 is expressed in skin-derived malignant T-cell lines, but not in non-malignant skin-derived T cells from MF lesions. While some malignant T cell lines express full-length wild-type CD22, others express wild-type and/or a novel CD22 splice variant. Analysis of CD22 and splice variant expression in CTCL lesions *in situ* revealed that the novel splice variant is expressed in 30% of the cases whereas only a few patients expressed wild-type CD22. In CD22-positive lesions, atypical T cells displayed co-expression of CD4 and CD22. Functional analysis indicates that both CD22 wild type and splice variants are involved in the regulation of the spontaneous proliferation of malignant T cells suggesting a role for CD22 in the pathogenesis of CTCL.

## RESULTS

### CD22 expression in malignant MF cell lines

**Figure 1:**
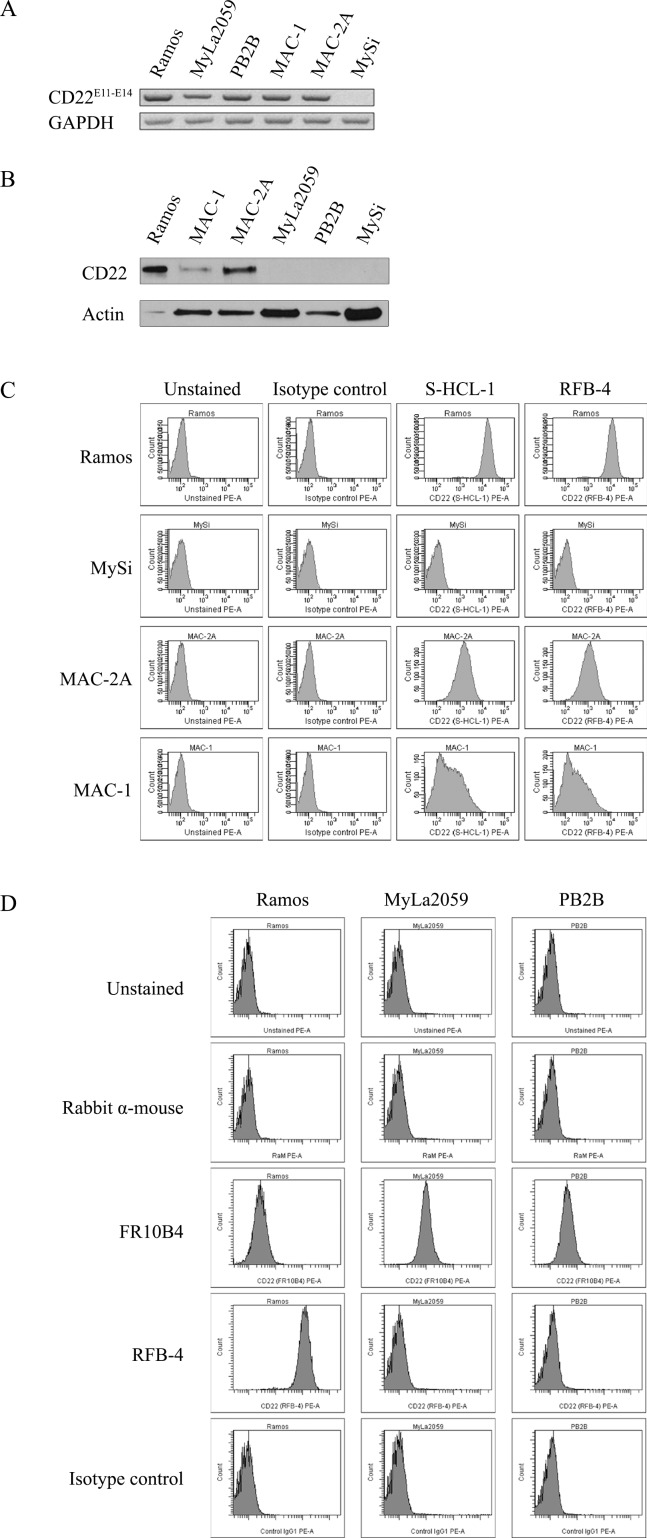
Expression of CD22 in CTCL cell lines **A**. RT-PCR analysis of CD22 expression using primers amplifying exon 11-14 region of CD22, **B**. Western blot analysis of CD22 protein expression using FPC1 anti-CD22 mAb, **C**. and **D**. Flow cytometry analysis of CD22 cell surface expression using PE-conjugated anti-CD22 mAbs (S-HCL-1, RFB-4) or unconjugated anti-CD22 mAbs (RFB-4, FR10B4) followed by PE-conjugated secondary antibody (rabbit anti-mouse F(ab')_2_).

To address whether malignant T cells express CD22, we initially performed RT-PCR analysis of CD22 expression using primers amplifying a region within exons 11-14 of CD22 in CTCL T lines, a non-malignant T cell line, and the Ramos B cells (as a positive control) [19]. As expected, the Ramos B cell line expressed CD22 mRNA (Fig. 1A, lane 1), whereas non-malignant T cells did not (Fig. 1A, lane 6). Surprisingly, all four malignant T cell lines expressed CD22 as judged from the RT-PCR analysis (Fig. 1A, lanes 2-5) indicating that malignant T cells may display ectopic expression of classic B cell markers in addition to BLK [4]. Next, we performed western blotting and flow cytometry analysis to address whether malignant T cells express CD22 protein of a correct size and whether CD22 is expressed as a surface protein similarly to the expression pattern in B cells. As shown by Western blot in Fig. 1B, the MAC2A cell line expressed high levels of CD22 protein (lane 3), the MAC-1 cell line expressed detectable but lower levels (lane 2), whereas the MyLa2059 and PB2B cell lines did not express detectable levels of CD22 protein (lanes 3 and 4). As expected, non-malignant T cells did not express CD22 protein (Fig. 1B, lane 5), whereas the Ramos B cell line expressed very high levels of CD22 protein (Fig. 1B, lane 1) as previously reported [19]. In parallel, the malignant T cell lines MAC-2A and MAC-1 (and the Ramos B cell line) showed membrane expression as detected by flow-cytometry using the anti-CD22 mAbs RFB-4 or S-HCL-1 (Fig. 1C), whereas non-malignant T cells (MySi, Fig. 1C) as well as MyLa2059 and PB2B did not (Fig. 1D, and data not shown). It should be noted, that the anti-CD22 mAb RFB-4 recognizes the extracellular domains 2-5 [20] while the mAb S-HCL-1 the N-terminal Ig domain [12]. Because CD22 splice variants have been described in B cell malignancies, we hypothesized that the apparent discrepancy between mRNA and protein expression of CD22 in some malignant T cells could be due to the expression of CD22 splice variants that are not recognized by the above-mentioned antibodies. Indeed, flow cytometry analysis using another anti-CD22 mAb, FR10B4, revealed a positive staining in all of the four malignant CTCL cell lines including the MyLa2059 and PB2B cell lines (Fig. 1D, and data not shown). These results demonstrate that some malignant T cell lines express the N-terminus or full length CD22 (CD22wt) and also suggest that other malignant T cells (MyLa2059 and PB2B) express an alternative variant of CD22 lacking the N-terminal portion of the protein.

### Expression of a novel splice variant of CD22 (CD22ΔN) in malignant T cells

**Figure 2:**
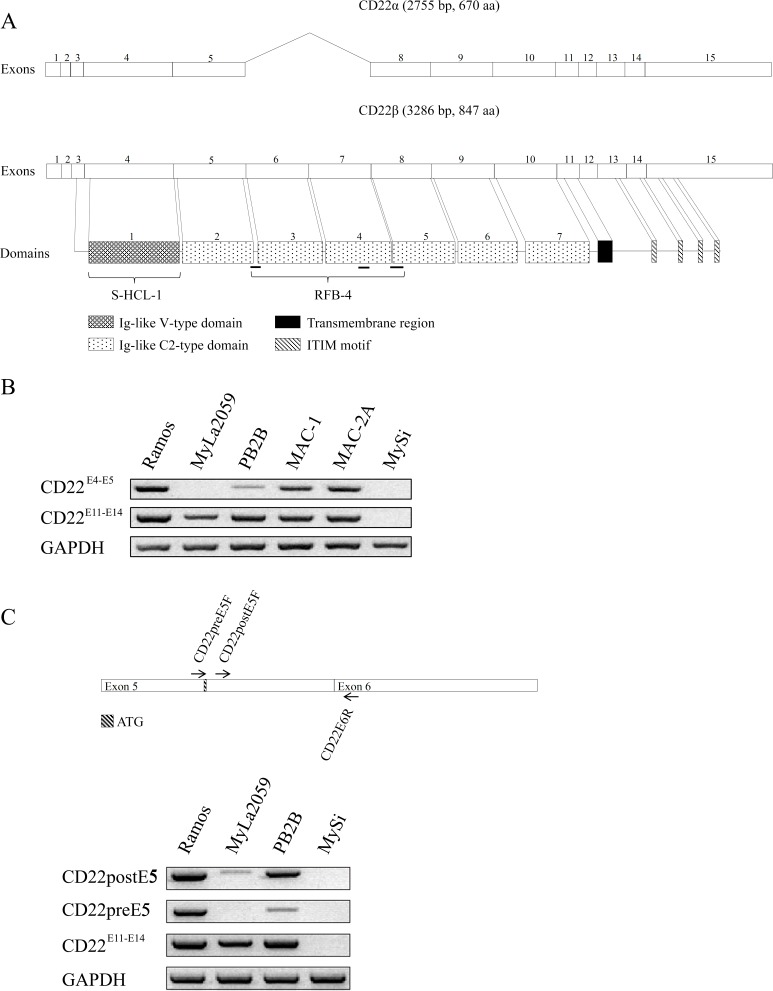
Expression of CD22 splice variants in a subset of MF cell lines **A**. Schematic representation of CD22 mRNA and protein structure, **B**. Exon-specific RT-PCR analysis of CD22 expression using primers amplifying exons 4-5 and/or exon 11-14 regions. C. RT-PCR analysis of CD22 expression using primers amplifying region before or after exon 5.

We next investigated whether the MyLa2059 and PB2B cell lines expressed alternative splice variants of CD22. Fig. 2A depicts a schematic representation of the CD22 gene, its domains, and known antibody relativities. Since the above cell lines demonstrated differential staining with antibodies against various forms of CD22 (Fig. 2A), we compared by RT-PCR the expression of the N-terminal (CD22^E4-5^) (recognized by antibody RFB-4) and the C-terminal (CD22^E11-14^) domains in malignant T cell lines, non-malignant T cells and the Ramos B cell line as control. As expected, Ramos B cells and the MAC-1 and MAC-2A cell lines expressed both the N- terminal (CD22^E4-5^) and C-terminal (CD22^E11-14^) domains (Fig. 2B) indicating that the cell lines express the full length CD22 (CD22wt) protein as also documented by the antibody recognition profiles (Fig. 1C).

Importantly, the MyLa2059 cell line expressed CD22^E11-14^ without expressing CD22^E4-5^ (Fig. 2B). Likewise, the MyLa2059 cell line tested negative for exons 3 and 4 using specific RT-PCR primers (data not shown). Taken together, these findings indicate that MyLa2059 cells transcribe the C-terminal- but not the N-terminal- domain of CD22. This conclusion is consistent with aforementioned observation that MyLa2059 cells express a CD22 variant protein (as determined by positive reactivity with antibody FR10B4), which lacks the N-terminal region. Hence, this variant of CD22 is not recognized by the RFB-4 or S-HCL-1 antibodies, both of which target the N-terminal part of CD22 (Fig. 1D and Fig. 2A). The PB2B cell line predominantly expressed the C-terminal (CD22^E11-14^) domain (Fig. 2B) but also weakly expressed the N-terminal domain (CD22^E4-5^) (Fig. 2B). Considering that exon 3 contains the start codon for the conventional CD22 protein (Fig. 2A), we hypothesized that CD22 (like CD33 [21]) has an alternative translation initiation site at position 624 that is used in MyLa2059 cells. Our findings indicate that these cells do not express CD22 mRNA upstream this alternative start codon, whereas detectable amplification occurred for regions downstream of the 624 positions start codon (Fig. 2C), suggesting that the translation of the alternative, novel CD22 splice form in MyLa2059 cell line might be initiated from a start codon in exon 5 instead of exon 3. This novel splice form of CD22 (hereby designated CD22ΔN) lacks the ligand-binding N-terminal domain of CD22 and thus differs from other known CD22 splice variants [12, 15, 16]. To test his hypothesis, we performed RT-PCR analysis of CD22 expression using primers amplifying region before or after exon 5. As shown in Fig. 2C (lower panel), MyLa2059 cells expressed low levels of CD22^postE5^ transcript, but not CD22^pre-E5^transcript. PB2B predominantly expressed CD22^postE5^ relative to the expression of the CD22^pre-E5^transcript (Fig. 2C), which was consistent with our findings presented in Fig. 2B. Taken together these results suggest that the PB2B T cell line expressed both the wild-type mRNA and the alternatively spliced variant (CD22ΔN) of the CD22 gene. Interestingly, only the splice variant appears to be expressed at the protein level as PB2B cells only stain positive with the FR10B4 mAb, but not with the RFB-4 and S-HCL-1 antibodies (Fig. 1D and data not shown).

### CD22 promotes survival and proliferation of malignant CTCL cells

**Figure 3:**
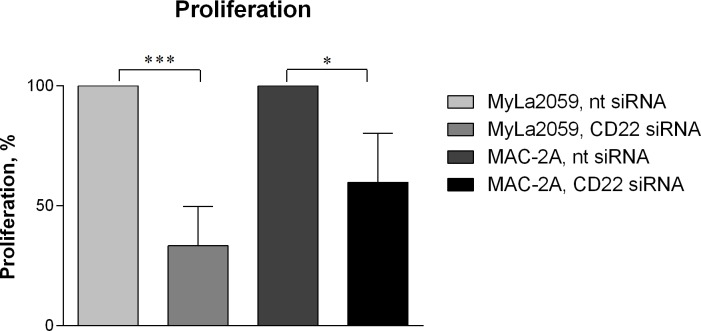
Silencing of CD22 MyLa2059 and MAC-2A cell lines were transfected with either non-targeting (−) or CD22-targeting (+) siRNA and cell proliferation were evaluated 24 hours after transfection. Results are representative of 5 independent experiments. Significant differences between non-targeting and CD22-targeting siRNA treated cells were evaluated using a paired two-tailed Student's t-test.

Having established that several malignant CTCL cell lines express CD22 (CD22wt and/or CD22ΔN), we next addressed their putative function in these cells. To achieve this, we silenced CD22 expression in MyLa2059 and MAC-2A cell lines using specific siRNA and evaluated the effect on the spontaneous growth 24 hours after siRNA transfection. As shown in Fig. 3, silencing of CD22 in CTCL cell lines significantly inhibited their proliferation when compared with cells treated with a non-targeting scrambled control siRNA (Fig. 3). The growth of MyLa2059 and MAC-2A cells were decreased by 67 % (p = 0.0008) and 40 %, respectively (p = 0.0118). Accordingly, silencing of CD22 expression resulted in a reduced number of viable cells (Supplementary Fig. S1). Taken together, these findings indicate that CD22ΔN and possibly CD22wt play an important role promoting cell survival and proliferation in CTCL.

### Ectopic expression of CD22 and CD22ΔN in skin lesions from CTCL patients

**Figure 4:**
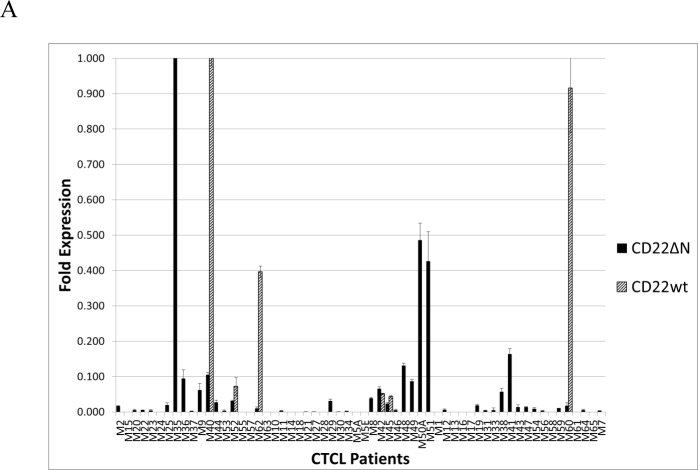

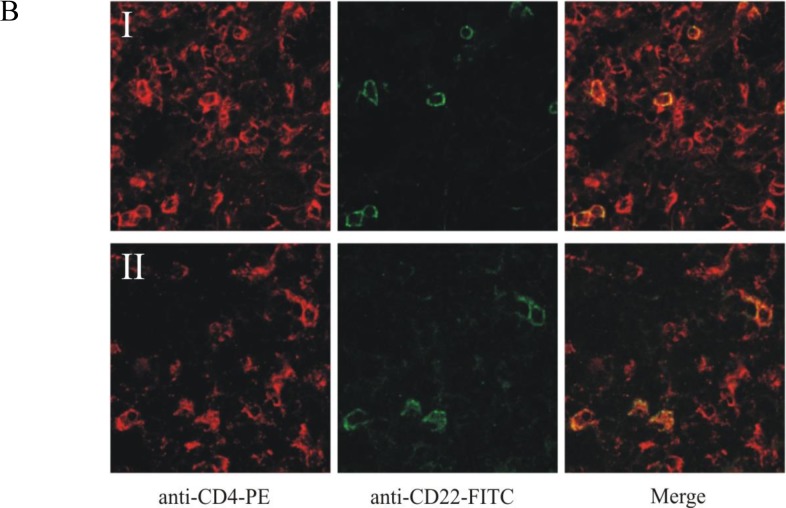
Expression of CD22 in patients with MF **A**. RT-PCR analysis of CD22 expression in CTCL lesional skin from patients. **B**. Double-staining with anti-CD4 (PE) and anti-CD22 (FITC) of biopsies from tumor stage of two MF patients.

To address whether CD22wt or CD22ΔN were expressed in lesional skin in CTCL patients, we tested for mRNA expression in a well-characterized cohort of 60 CTCL patients [22-27]. Using the primer set specifically recognizing CD22ΔN, 39 out of 60 (65%) of CTCL patients expressed this variant of CD22 (Fig. 4A), while only 6 out of 60 patients (10%) were positive for CD22wt (Fig. 4A) indicating that a substantial number of patients display expression of CD22ΔN mRNA in lesional skin, whereas expression of CD22wt was relatively rare (10%) among CTCL patients. Only three patients co-expressed CD22wt and CD22ΔN, three expressed CD22wt but not CD22ΔN, while 15 expressed CD22ΔN but not CD22wt (Fig. 4A). This finding mirrors the observation of CD22 expression in immortalized malignant CTCL cell lines, where all of these genotypes are also present.

Since FR10B4 monoclonal antibody is not suitable for immunohistochemistry (data not shown), we were not able to determine whether CD22ΔN is expressed as protein in CTCL lesional skin. However, we were able to test the expression of the CD22 wild type variant in immunohistochemistry sections from patients. Furthermore, an important question arises, whether the observed expression is due to CD22 expression by malignant T cells or by infiltrating by-stander B cells? To answer this, we performed dual staining for CD22 and CD4 in CTCL lesional skin. In an independent set of 14 MF patients, for which fresh tissue biopsies were available, we identified strong CD22wt expression in 2 patients, whereas one patient was borderline positive (data not shown) indicating a frequency (7-12%) comparable to that found for CD22wt by RT-PCR in the Boston cohort of CTCL patients (Fig. 4A). Dual staining analysis in these 2 patients demonstrated strong co-localization of CD4 and CD22wt signals in the same malignant cells (Fig. 4B right). Notably, the double positive T cells were often found in small clusters, (e.g. some areas were completely empty, whereas up to 30% of the CD4-positive cells co-stained with CD22 in other areas) suggesting a heterogeneous CD22wt expression in these tumors. Taken together these results suggest that CD22 is expressed in a subset of patients both at as mRNA and protein levels.

## DISCUSSION

Malignant transformation is known to cause aberrant gene expression resulting in ectopic upregulation of proteins that are usually expressed in other cell types [28-31]. In the current work we demonstrate that CD22, a sialic acid-binding lectin previously exclusively described in B cells, is expressed in malignant T cell lines derived from skin lesions of MF patients (MAC-1, MAC-2A, MyLa2059 and PB2B) and *in situ* in lesional skin from CTCL patients. CD22 expression was verified both at the mRNA and protein levels, including the co-expression of CD22 and CD4 in MF lesions *in situ*. Two cell lines, MyLa2059 and PB2B, were lacking CD22wt reactivity with two specific mAbs, S-HCL-1 and RFB-4 recognizing epitopes located in N-terminal domain of CD22, despite expression of CD22 mRNA, suggesting that these cells express an alternative splice variant of CD22. This was confirmed by exon-specific RT-PCR demonstrating that MyLa2059 cells do not express exon 4 and part of exon 5; these exons correspond to the ligand binding domains of CD22. RT-PCR using primers specific for sequences upstream or downstream of a potential start codon in exon 5 demonstrated that translation of the shorter isoform of CD22 may be initiated from a start codon in exon 5 instead of exon 3. RT-PCR also demonstrated that PB2B cells express both the conventional and the splice variant of CD22 lacking the N-terminal domain and accordingly designated CD22ΔN. Since the alternative sequence does not include a targeting signal peptide, one might question how CD22 is able to get targeted to the cell surface and whether the transport of CD22 to the cell surface is mediated via either co-transporter protein(s), or other unknown mechanisms. The phenomenon is, however, not unprecedented as a recently described isoform of CD33, another member of the Siglec family of lectins, also lack a ligand-binding domain. Yet, it is highly expressed in human lymphoid and myeloid cell lines as well as in monocytes, neutrophils and activated T cells obtained from the blood of healthy donors [32]. Therefore it is intriguing whether a similar splicing switch occurs in the case of CD22. In particular, since this isoform of CD33 is co-expressed with the conventional CD33 [32], which parallels our findings of residual expression of exons 4 and 5 in the PB2B cell line.

RT-PCR analysis of lesional skin indicates that CD22ΔN and/or CD22wt are expressed in MF lesions up to 60% of tested patients, suggesting that an aberrant CD22ΔN/CD22wt expression might be important in the pathogenesis of CTCL. Due to a lack of CD22ΔN reactive antibodies for IF/IHC, we were only able to confirm the expression of CD22wt protein *in situ* in approximately 10% of CTCL patients, i.e. the same frequency as detected by mRNA expression. Importantly, CD22wt was co-expressed with CD4 confirming that it is indeed expressed by T cells in the MF lesions, demonstrating that T cells express CD22wt in MF lesions *in situ* arguing against the possibility that an ectopic CD22 expression is merely a reflection of an infiltration of B cells.

The observation that siRNA-mediated CD22 silencing results in a profound inhibition of the survival and proliferation in malignant T cell lines further supports the notion, that CD22 exerts a functional role in the pathogenesis of CTCL and as such, may be a novel target for therapy. Toxin-coupled anti-CD22wt antibodies are currently being examined for therapeutic efficacy in the treatment of B cell malignancies, and if successful, these reagents may also be useful in patients with CD22wt CTCL [33]. The generation of toxin-coupled antibodies with specificity for CD22ΔN or broadly, cross-reacting antibodies recognizing both CD22ΔN, CD22wt, (and possibly other splice forms) would be even more relevant in future antibody mediated therapies for both CTCL and B cell malignancies.

In conclusion, we provide the first data demonstrating that CD22wt and the new CD22 splice form lacking the N-terminal domain (CD22ΔN) are expressed in 4 CTCL cell lines and in two third of MF lesions. Functional studies indicate that CD22ΔN/CD22wt promote cell survival and proliferation suggesting a pathogenic role of CD22 in CTCL.

## MATERIALS AND METHODS

### Antibodies

FR10B4, FPC1 and PE-conjugated or unconjugated RFB-4 mouse anti-CD22 monoclonal antibodies (mAbs) as well as negative control Q13 mouse anti-IL-12A p35 mAb were purchased from Santa Cruz Biotech. (Santa Cruz, CA, USA). PE-conjugated S-HCL-1 anti-CD22 mAb was purchased from Becton Dickinson (San Jose, CA, USA). AC-40 mouse anti-Actin mAb was purchased from Sigma-Aldrich (St-Louis, MO, USA). HRP-conjugated rabbit anti-mouse immunoglobulins and rabbit anti-mouse PE-conjugated F(ab')2 immunoglobulins were purchased from Dako (Glostrup, Denmark).

### Cell lines and cell culture

The malignant T-cell lines, MAC-1, MAC-2A, MyLa2059, PB2B and the non-malignant tumor infiltrating T-cell line, MySi, were established from patients diagnosed with MF, and has previously been described [34-36]. Ramos 2G6.4C10 malignant B-cell line derived from a Burkitt lymphoma has been described elsewhere [37]. Cell lines were cultured in RPMI-1640 supplemented with 2 mM L-glutamine, 100 μg/ml penicillin/streptomycin (Sigma-Aldrich, St-Louis, MO, USA) and 10% heat-inactivated fetal bovine serum (FBS) (Life Technologies, Roskilde, Denmark). MySi cell line was cultured in 10% pooled human serum (Blood Bank, State University Hospital, Copenhagen, Denmark) instead of 10% FBS and was additionally supplemented with 103 U/ml IL-2 (Proleukin, Chiron, Emeryville, CA, USA).

### Patients and samples

The frozen tissue samples used for immunofluorescence were obtained from patients that were enrolled in an ethic committee-approved study protocol with informed consent (Ethics Committee of the Medical Faculty of the University of Würzburg, handling code 135/06 and 162/06). All tissue samples were obtained and processed as previously described [4]. All tissue samples used for analysis of CD22 expression were from patients enrolled in an IRB-approved study protocol with informed consent. The historic cohort of patients from Boston has been described previously in multiple publications [22-27]. All tissue samples were obtained and processed as previously described [25, 26]. Briefly, 6 mm punch biopsies from involved skin were collected from CTCL patients at the time of the initial diagnosis between January 26, 2003 and June 1, 2005. The obtained 6 mm biopsies were immediately snap-frozen in liquid nitrogen. Tissue was powdered in liquid nitrogen (Cryo-Press; Microtec Co, Chiba, Japan), and total RNA was extracted using Trizol (Invitrogen, Carlsbad, CA) and converted to cDNA using the iScript RT-PCR kit (Bio-Rad, Hercules CA) according to the manufacturer's instructions. The biopsy samples analyzed in this report are the same samples that were previously analyzed elsewhere [22-27].

### Immunofluorescence

For tissue samples, frozen sections were dried overnight and fixed in acetone followed by blocking with 1% BSA/PBS. All subsequent incubation steps were performed at room temperature for 30min followed by two washes with 1% BSA/PBS. Slides were incubated with the anti-CD22wt antibody RFB-4 (1:250) followed by a FITC-conjugated anti-mouse IgG (Dianova; Hamburg, Germany), and finally a PECy5-conjugated anti-CD4 antibody (1:500, clone MT310, Millipore/Merck KGaA, Darmstadt; Germany). Slides were mounted in vectashield and analyzed with a Leica confocal microscope.

### RNA purification and reverse transcriptase-PCR

Total cellular RNA was purified using the RNeasy RNA purification kit with the addition of on-column DNase digestion to avoid DNA contamination as described by the manufacturer (Qiagen; Hilden, Germany). Purified RNA was reverse transcribed using murine Moloney leukemia virus reverse transcriptase (M-MLV, Invitrogen) and oligo(dT) primer. The resulting cDNA was amplified by PCR using recombinant Taq DNA polymerase (no. M0267; New England Biolabs, Beverly, MA, USA) with the following primers: GAPDH forward: 5′-CCATGGAGAAGGCTGGGG, GAPDH reverse: 5′-CAAAGTTGTCATGGATGACC; CD22 preE5 forward: 5′-CTCCTAGAGGGGGTTCCAAT, CD22postE5 forward: 5′-GTCACCTCGACCTCCTTGAC, CD22E6 reverse: 5′-GGGAGTGACCTTGATCTCCA; CD22E4 forward: 5′-TTTTTGAGCACCCTGAAACC, CD22E5 reverse: 5′-CGGATACCCATAGCAGGAGA; CD22E11 forward: 5′-CATCCTCATCCTGGCAATCT, CD22E14 reverse: 5′-CTCTGCATCTCCAGTTCGTG. PCR program as follows: 94 ºC for 2 min, 32 cycles of 94 ºC for 15 s, at 56 ºC for 30 s, at 72 ºC for 30 s, and finally at 72 ºC for 10 min. The samples were analyzed in a 3 % agarose gel. The expression of CD22 in CTCL skin biopsy samples was analyzed using the above primers. The obtained expression results were standardized using genorm method utilizing ACTB, SDHA and B2M housekeeping genes [38].

### Protein extraction and western blotting

Cells were rapidly pelleted and lysed in ice-cold lysis buffer and subjected to sodium dodecyl sulfate-polyacrylamide gel electrophoresis and western blotting as previously described [39]. Membranes were blotted with mouse anti-CD22 mAb, FPC1 (Santa Cruz Biotech., Santa Cruz, CA, USA), and AC-40 anti-Actin mAb (Sigma-Aldrich, St-Louis, MO, USA) followed by rabbit anti-mouse Igs-HRP (Dako, Glostrup, Denmark).

### Flow cytometry

For surface staining, cells were harvested, washed in ice-cold FACS buffer (5 % FBS and 0.1 % Na-azide in PBS) and stained with CD22-specific mAbs or matched isotype controls (1:50) for 30 min at 4ºC in darkness. When indicated, staining with unconjugated primary antibody was followed by staining with PE-conjugated rabbit anti-mouse Igs (F(ab')2) (1:400). After final washes, cells were resuspended in FACS buffer and analyzed on a BD LSRII (BD Biosciences) flowcytometer equipped with FACSDiva software.

### siRNA transfection

CD22 was silenced by transfecting cells with 0.5 nmol of Accell SMART pool® CD22-targeting siRNA from Dharmacon (Lafayette, CO, USA). SiGENOME non-targeting siRNA pool #1 from Dharmacon was used as a scrambled control. Cells were transfected with an Amaxa nucleofector (Amaxa, Cologne, Germany) as described previously [40]. Cells were immediately transferred to a 6-well culture plate containing fresh growth medium without antibiotics. Transfected cells were used for proliferation and viability assays.

### Proliferation assay

Cell proliferation was evaluated via [methyl-3H]-thymidine (Amersham, Hillerød, Denmark; 7 μCi/sample) incorporation assay as described elsewhere [41].

### Cell viability assay

Viability was determined with a MTT-assay as described previously [35]. Light absorbance at 570 nm wavelength was measured by scanning the plates with Multiskan FC microplate photometer (Thermo Fisher Scientific).

## SUPPLEMENTARY MATERIAL FIGURE



## Abbreviations

CD22: cluster of differentiation 22
BLK: B-lymphoid kinase
CTCL: cutaneous T-cell lymphoma
Ig: immunoglobulin
MF: Mycosis Fungoides
siRNA: small interfering RNA.
