# Supplementary material for: Ectopic expression of a novel CD22 splice-variant regulates survival and proliferation in malignant T cells from cutaneous T cell lymphoma (CTCL) patients
Source: Oncotarget. 2015 Mar 30;6(16):14374–84. doi: 10.18632/oncotarget.3720 (PMC4546473; doi:10.18632/oncotarget.3720)
Supplement: Supplementary file 1 [file oncotarget-06-14374-s001.pdf]

Ectopic expression of a novel CD22 splice-variant regulates survival and proliferation in malignant T cells from cutaneous T cell lymphoma (CTCL) patients

Supplementary Material

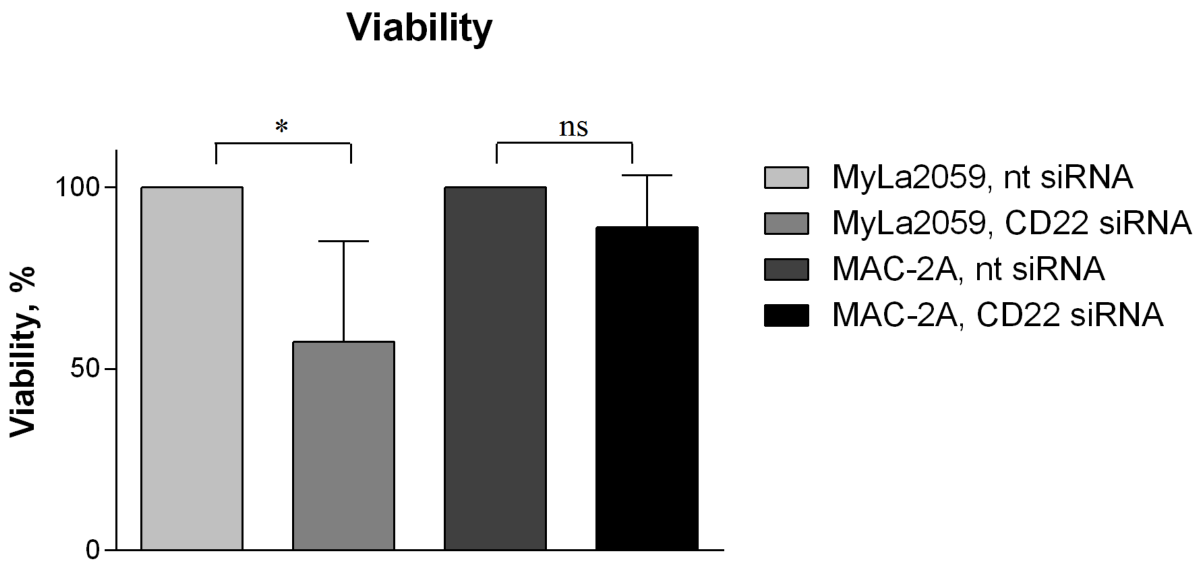

Supplementary Figure 1: Silencing of CD22. MyLa2059 and MAC-2A cell lines were transfected with either non-targeting (-) or CD22-targeting (+) siRNA and cell viability were evaluated 24 hours after transfection in a MTT assay. Results are representative of 5 independent experiments. Significant differences between non-targeting and CD22-targeting siRNA treated cells were evaluated using a paired two-tailed Student's t-test.
